# A phospholipase D2 inhibitor, CAY10594, ameliorates acetaminophen-induced acute liver injury by regulating the phosphorylated-GSK-3β/JNK axis

**DOI:** 10.1038/s41598-019-43673-x

**Published:** 2019-05-10

**Authors:** Sung Kyun Lee, Geon Ho Bae, Ye Seon Kim, Hyung Sik Kim, Mingyu Lee, Jaewang Ghim, Brian A. Zabel, Sung Ho Ryu, Yoe-Sik Bae

**Affiliations:** 1 Department of Biological Sciences, Sungkyunkwan University, Suwon, 16419 Republic of Korea; 2 Department of Health Sciences and Technology, SAIHST, Sungkyunkwan University, Seoul, 06351 Republic of Korea; 3 Department of Life Science, POSTECH, Pohang, 37673 Republic of Korea; 4 Palo Alto Veterans Institute for Research, Veterans Affairs Hospital, Palo Alto, CA 94304 USA; 5 Present Address: Institute for Stem Cell & Regenerative Medicine Research of Albert Einstein College of Medicine, Bronx, NY 10461 USA

**Keywords:** Target identification, Molecular medicine

## Abstract

We examined the role of phospholipase D2 (PLD2) on acetaminophen (APAP)-induced acute liver injury using a PLD2 inhibitor (CAY10594). 500 mg/kg of APAP challenge caused acute liver damage. CAY10594 administration markedly blocked the acute liver injury in a dose-dependent manner, showing almost complete inhibition with 8 mg/kg of CAY10594. During the pathological progress of acute liver injury, GSH levels are decreased, and this is significantly recovered upon the administration of CAY10594 at 6 hours post APAP challenge. GSK-3β (Serine 9)/JNK phosphorylation is mainly involved in APAP-induced liver injury. CAY10594 administration strongly blocked GSK-3β (Serine 9)/JNK phosphorylation in the APAP-induced acute liver injury model. Consistently, sustained JNK activation in the cytosol and mitochondria from hepatocytes were also decreased in CAY10594-treated mice. Many types of immune cells are also implicated in APAP-induced liver injury. However, neutrophil and monocyte populations were not different between vehicle- and CAY10594-administered mice which are challenged with APAP. Therapeutic administration of CAY10594 also significantly attenuated liver damage caused by the APAP challenge, eliciting an enhanced survival rate. Taken together, these results indicate that PLD2 is involved in the intrinsic response pathway of hepatocytes driving the pathogenesis of APAP-induced acute liver injury, and PLD2 may therefore represent an important therapeutic target for patients with drug-induced liver injury.

## Introduction

Although acetaminophen (APAP), an over-the counter drug, is widely used as an analgesic and antipyretic, overdose may cause serious hepatotoxicity due to hepatocellular necrosis^1,2^. Drug-induced liver injury (DILI) is a major problem caused by APAP overdose, and DILI accounts for more than 50% of acute liver failure in the United States^1^. Accumulation of a reactive metabolite of APAP, N-acetyl-p-benzoquinone-imine (NAPQI), in hepatocytes stimulates the generation of intracellular reactive oxygen species and subsequent mitochondrial dysfunction and DNA damage^3^. In overdose conditions, oxidation by cytochrome P450 enzymes becomes important in the metabolism of APAP, and excess NAPQI binds to SH- groups in cellular proteins, causing cell injury^4^. During this process, the glutathione (GSH) supply is exhausted^5^. GSH depletion and covalent binding of mitochondrial proteins were traditionally thought to directly lead to mitochondrial dysfunction by triggering mitochondrial permeability transition, resulting in hepatocyte death and liver injury. Recent studies suggest that GSH depletion and impaired mitochondria function can lead to release of reactive oxygen species that subsequently can activate c-Jun N-terminal kinase (JNK), a member of the MAPK family^6^. Activated JNK translocates to the mitochondria, which leads to mitochondrial dysfunction through mitochondrial permeabilization and cytochrome c release^7,8^. Recent reports have shown that GSK-3β is an important mediator causing APAP-induced liver injury^9^. However, the detailed molecular mechanism involved in APAP-induced DILI remains unclear.

Phospholipase D (PLD) is an important lipid-hydrolyzing enzyme that specifically hydrolyzes phosphatidylcholine to phosphatidic acid (PA) and choline^10^. Two different isoforms of mammalian PLD, PLD1 and PLD2, are reported to carry two HKD catalytic motifs, one PX and one PH domain^10,11^. PLD activity is regulated by several molecules including phosphoinositide and protein kinase C^10,11^. Regarding its physiological role, PLD has been reported to regulate many different cellular processes including cell proliferation, apoptosis, cell differentiation, vesicle transport, and cell migration^12–14^. Recent reports demonstrated that PLD2 mediates the hypoxic response and pathological angiogenesis^15^ and septic shock in response to bacterial infection^16^. Very recently, it has been reported that phosphatidic acid (PA), a product of PLD enzymatic activity, is elevated during APAP-induced acute liver injury and APAP overdose patients^17^. However, a functional role for PLD, especially PLD2, in the pathogenesis of DILI is yet to be investigated. In this study, we examined the role of PLD2 on DILI and the related mechanism of action using a PLD2-selective inhibitor.

## Results

### CAY10594 administration blocks liver damage of the APAP overdose-induced acute liver injury model

**Figure 1:**
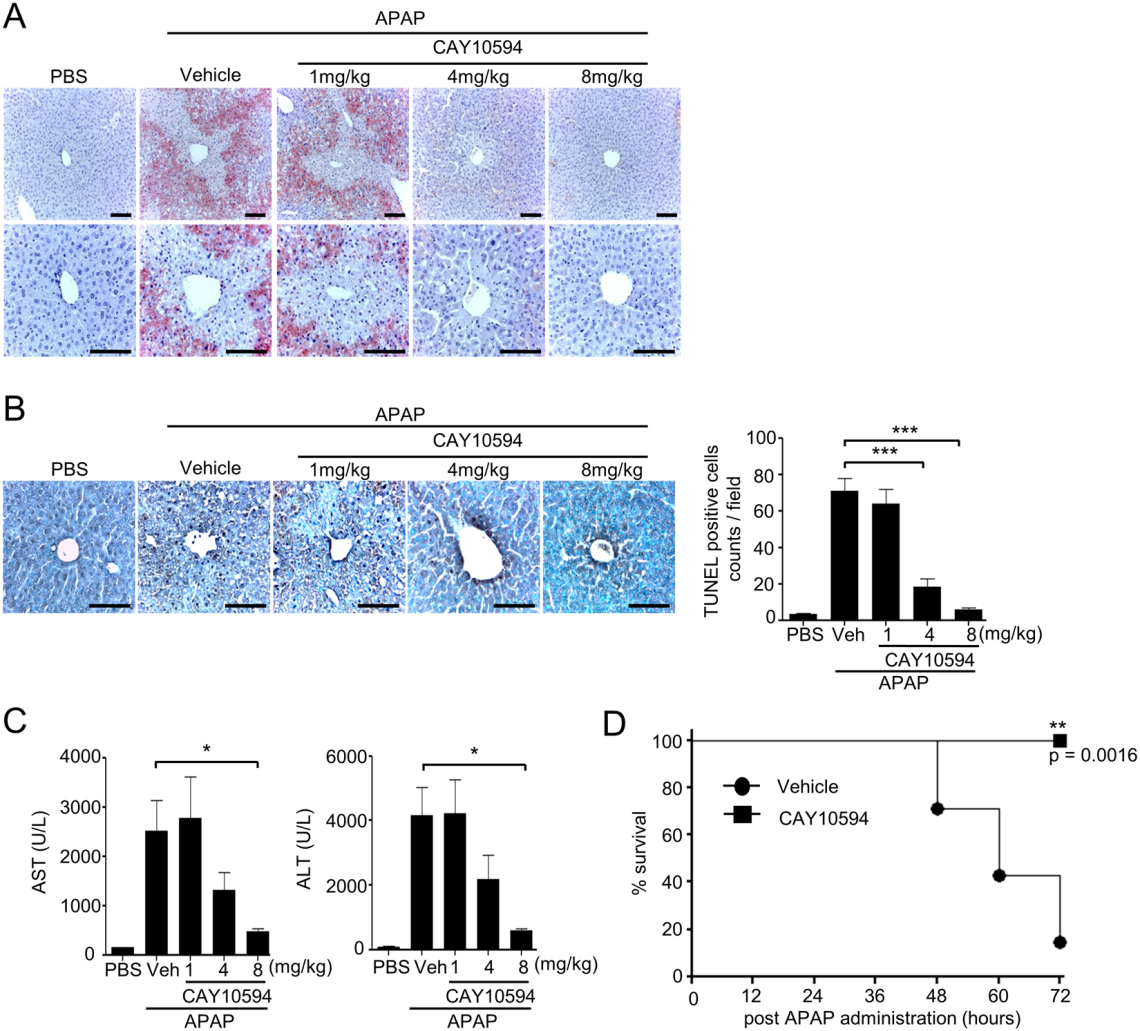
CAY10594 attenuates APAP overdose-induced acute liver injury. (**A**–**C**) Mice were orally challenged with APAP (500 mg/kg) alone or with various concentrations of CAY10594 (1 to 8 mg/kg) at 1 h before APAP injection and were sacrificed 12 h later. (**A**) Livers were stained with hematoxylin and eosin (magnification, x200 or x400). (**B**) Apoptotic cells were visualized as measured by TUNEL histology apoptotic cells were counted at x200 magnification. Scale bar, 100μm (A, B left). (C) AST and ALT levels in sera, which was measured at 12 h after APAP treatment. (**D**) Lethal dose APAP (750 mg/kg)-induced mortality was monitored every 12 h for 72 h. Data are expressed as the mean ± SEM (n = 5 for **B**,**C**, n = 7 for **D**). *P < *0.05*; by *t*-test compared with treatment of APAP in vehicle control mice (**B**,**C**). Survival was analyzed by log-rank test. **P < *0.01* (**D**). Data are representative of two independent experiments.

At first, we investigated the role of PLD2 on acute liver injury using a CAY10594. Injection of APAP (500 mg/kg) caused marked liver injury, which was measured by hematoxylin and eosin staining of the livers (Fig. 1A). APAP-induced liver injury was almost completely blocked by the administration of a CAY10594 in a dose-dependent manner (Fig. 1A). APAP-induced hepatocyte death was measured by the TUNEL assay. Hepatocyte apoptosis was induced by APAP, which was also markedly decreased in CAY10594-administered mice compared with vehicle-treated mice (Fig. 1B). The protective effects of the CAY10594 against hepatocyte apoptosis were strongly induced at 4 or 8 mg/kg (Fig. 1B).

We then measured aspartate aminotransferase (AST) and alanine aminotransferase (ALT) activity in serum collected from APAP + vehicle or APAP + CAY10594 administered groups. Injection of APAP caused marked increase in both AST and ALT levels in serum, which were significantly decreased by CAY10594 in dose-dependent manners (Fig. 1C). Administration of 8 mg/kg CAY10594 almost completely blocked the increase of AST and ALT levels in APAP mice serum (Fig. 1C). However, PLD1 inhibitor (VU0155069) injection did not block increased AST and ALT levels upon APAP administration (data not shown). A lethal dose of APAP (750 mg/kg) elicited severe mice mortality within 72 hours (Fig. 1D). However, administration of CAY10594 significantly blocked the mortality, showing 100% survival for 72 hours (Fig. 1D). Taken together, these results indicate that PLD2 inhibitor, but not PLD1 inhibitor, shows strong protective effects against APAP-induced acute liver injury.

### CAY10594 treatment causes rapid recovery of APAP-induced decreased GSH levels but decreases the sustained activation of JNK in the liver

**Figure 2:**
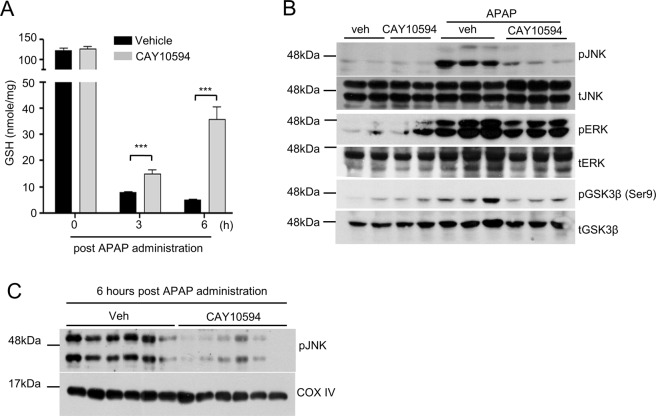
Effects of PLD2 inhibition on APAP-induced depletion of GSH levels and JNK activation in the liver. (**A**–**C**) Vehicle or CAY10594 was administered 1 h before APAP treatment (500 mg/kg). (**A**) Total GSH from liver tissues was determined using a commercial GSH assay kit (Enzo Life Sciences Inc, Farmingdale, NY, USA). (**B**,**C**) Phosphorylation of JNK, ERK, GSK-3β and total JNK, ERK, GSK-3β from whole liver tissue lysates or (**C**) a mitochondria fraction was determined by Western blot analysis. Data are expressed as the mean ± SEM (n = 5 for **A**). ***P < *0.001*; by *t*-test compared with the vehicle treated group with APAP administration (**A**). Data are representative of two independent experiments.

GSH levels have been previously reported to be decreased during acute liver injury^18^. Administration of an overdose of APAP rapidly depletes GSH from the liver of mice^18^. We also observed that GSH levels were strongly decreased at 3 or 6 hours post APAP challenge (Fig. 2A). However, GSH levels were markedly recovered in CAY10594 administered mice at 3 or 6 hours after the APAP challenge (Fig. 2A). Presumably, inhibition of PLD2 could repair liver injury or block hepatic cell death signaling during APAP consumption in the DILI mouse model.

JNK phosphorylation has been reported to be mainly associated with increased acute liver injury^8^. In this study, we also found that the administration of 500 mg/kg APAP strongly induced JNK phosphorylation in the liver of vehicle-injected mice (Fig. 2B). At 2 hours post APAP challenge, JNK phosphorylation was apparent in the entire liver tissue, and this was markedly attenuated by CAY10594 treatment (Fig. 2B). In addition to JNK, extracellular signal-regulated kinase (ERK) is also known as a key mediator of inflammation and oxidative stress. Inhibition of ERK activation can attenuate APAP-induced liver injury^19^. Therefore, we investigated the role of PLD2 on APAP-induced MAPK phosphorylation. APAP treatment promoted the levels of phosphorylated-ERK in vehicle-injected mice (Fig. 2B). However, APAP-induced ERK phosphorylation was significantly attenuated by CAY10594 treatment (Fig. 2B).

Since phosphoryated GSK-3β mediates the early phase of APAP-induced liver injury, we examined the effects of CAY10594 on the phosphorylation of GSK-3β, which triggers mitochondrial dysfunction in the liver. CAY10594 significantly blocks APAP-induced GSK-3β phosphorylation at 2 hours post APAP administration (Fig. 2B).

Sustained activation of JNK in the cytosol can lead to translocation of activated JNK to the mitochondria, which is associated with the initiation of mitochondrial dysfunction that can lead to hepatocyte necrosis^8,20^. As expected, inhibition of PLD2 led to blockage of phosphorylated-JNK translocation to the mitochondria (Fig. 2C). The results suggest that inhibition of PLD2 could prevent mitochondrial dysfunction by inhibiting JNK translocation. Collectively, inhibition of PLD2 may show protective effects against acute liver injury by attenuating JNK phosphorylation in the APAP challenge.

### CAY10594 administration regulates cytokine and chemokine production but not immune cell recruitment after APAP overdose

**Figure 3:**
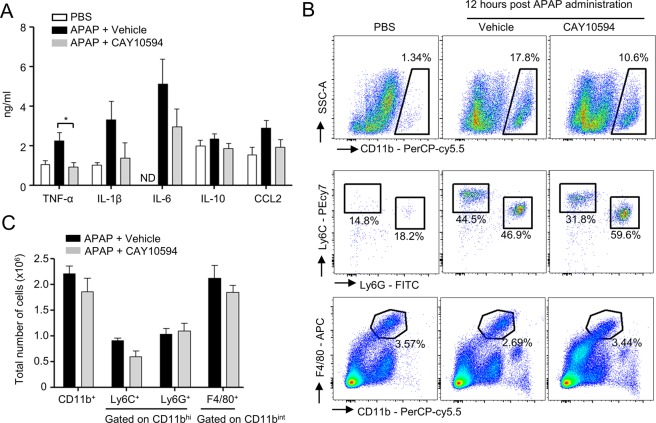
Effects of CAY10594 on cytokine production and distribution of immune cells in liver after APAP overdose. (**A**–**C**) Vehicle or CAY10594 was administered by intraperitoneal injection 1 h before APAP treatment (500 mg/kg). (**A**) Peripheral blood was collected at 12 h after the APAP challenge. The amounts of cytokine in the serum were determined by ELISA analysis. (**B**,**C**) Primary cells from liver tissue were isolated using perfusion with collagenase. These cells were stained against CD11b-PerCP/cy5.5, Ly6G-FITC, Ly6C-PE/cy7, F4/80-APC and analyzed by flow cytometry. Data are expressed as the mean ± SEM (n = 5–6 for **A,C**). (**A**) ND = Not detected, (**A**,**C**) *P < *0.05*; by *t-*test compared with the vehicle treated group with APAP administration (**A**). Data are representative of two independent experiments.

Overdose drug-induced liver injury is caused by cytosolic reactive oxygen species and intrinsic signaling molecules, GSK-3β, JNK and mitochondrial reactive oxygen species, which subsequently induce hepatic cell death^9^. Extrinsic signaling mediators, pro-inflammatory cytokines, and damage-associated molecular patterns, have been also closely associated with APAP-induced liver failure^21^. In this study, we tested the effects of the CAY10594 on the expression levels of inflammatory cytokines and chemokines in APAP-induced acute liver injury. The administration of 500 mg/kg APAP strongly increased the levels of TNF-α, IL-6, IL-1β, and CCL2 in the sera from the vehicle administered group. However, the increase of pro-inflammatory cytokines and chemokine by APAP were restrained by treatment with CAY10594 (Figs. 3A).

Many types of immune cells are largely involved in hepatic injury through the secretion of pro- or anti-inflammatory cytokines such as TNF-α, IL-6, IL-1β and IL-10^22^. These cytokines serve to promote inflammation during APAP-induced hepatotoxicity^22^. Here, we investigated the effects of CAY10594 on the composition of immune cell population in the liver of APAP-challenged mice. The total number of myeloid cells, including neutrophils (CD11b^hi^ Ly6G^+^), mononuclear cells (CD11b^hi^ Ly6C^+^), and Kupffer cells (CD11b^int^ F4/80^hi^) showed no significant difference in both vehicle- and CAY10594-treated mice after the APAP challenge (Fig. 3B,C).

### CAY10594 shows decreased APAP-induced hepatotoxicity *in vitro*

**Figure 4:**
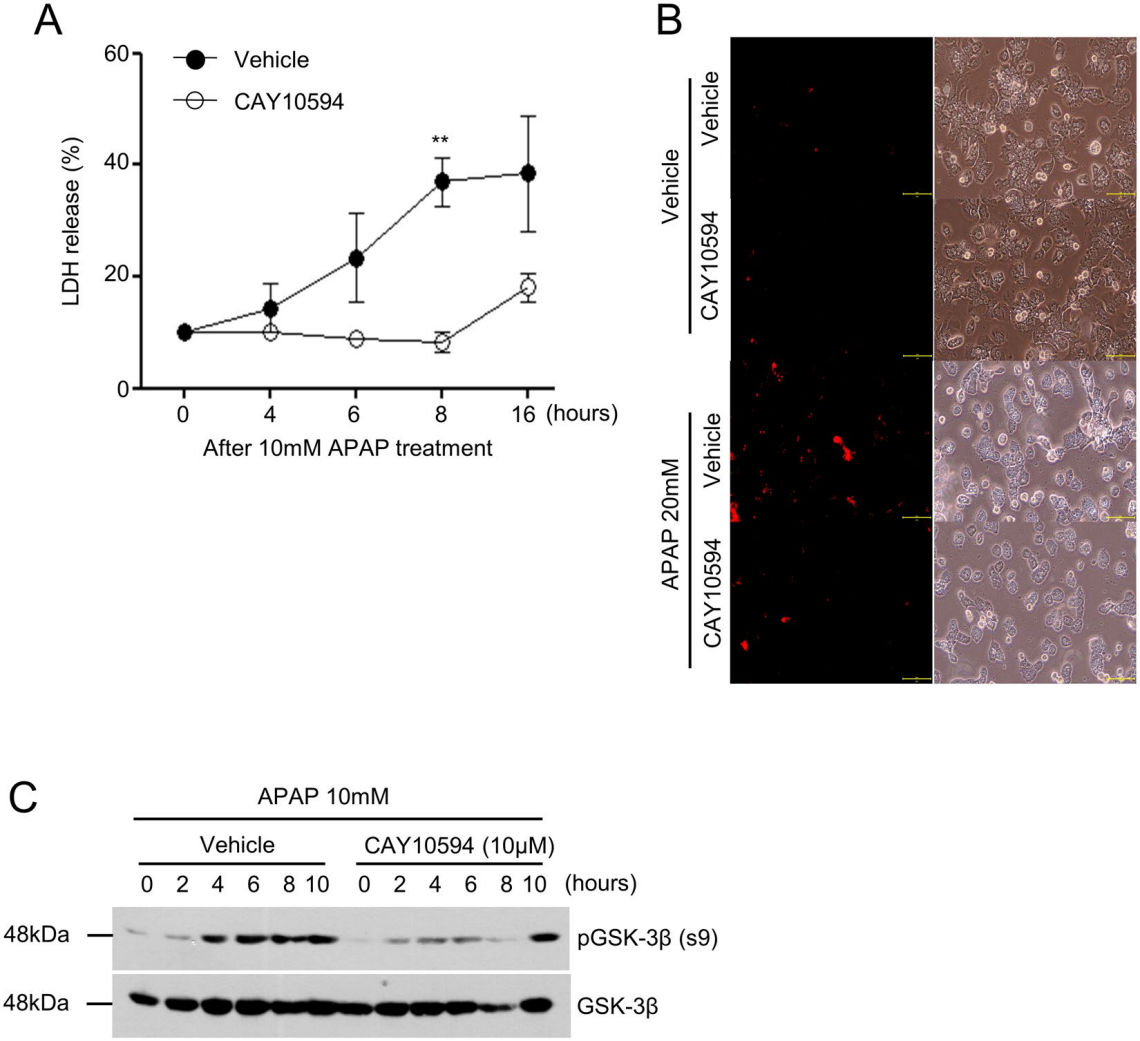
CAY10594 blocks APAP-induced primary hepatocyte death. (**A**–**C**) Primary hepatocytes were isolated from mice liver using collagenase. Cells were preincubated with CAY10594 (10 μM) or not in the absence or presence of 10 mM APAP in a time-dependent manner. (**A**) LDH activity in the supernatant was assessed by the LDH cytotoxicity assay kit, and OD values at 490 nm are presented as a line graph. (**B**) To determine hepatocyte death, hepatocytes were stained against propidium iodide (PI) and the number of PI positive cells were counted at x200 magnification by fluorescence microscopy. Scale bar, 100μm (B). (**C**) Phosphorylation of GSK-3β and total GSK-3β from primary hepatocytes was determined by Western blot analysis. All data are represented as the mean ± SEM. The data are representative of two independent experiments. **P < *0.01;* by *t*-test compared with APAP-treated cells given the vehicle (**A**).

Acute liver injury can be induced by hepatocyte apoptosis in response to APAP challenge *in vitro*. In this study, we tested the effects of CAY10594 on hepatocyte toxicity in response to APAP in primary hepatocytes. Isolated primary hepatocytes were stimulated with 10 mM APAP for several lengths of time. We measured LDH release, which is a biomarker for cytotoxicity in damaged cells. Hepatocytes were found to be damaged by APAP after 4 hours, and approximately 40% of the cells were damaged at 16 hours. However, CAY10594 treatment significantly decreased the APAP-induced hepatocyte damage (Fig. 4A). The addition of APAP into hepatocytes markedly increased the death of hepatocytes showing PI^+^ cells, which were decreased by CAY10594 treatment (Fig. 4B). *In vitro* stimulation of primary hepatocytes with APAP (10 mM) also induced GSK-3β phosphorylation (Fig. 4C). Addition of CAY10594 markedly blocked APAP-induced GSK-3β phosphorylation in primary hepatocytes (Fig. 4C). Taken together, the results suggest that PLD2 plays a key role in the intrinsic response pathway of hepatocytes to APAP-induced hepatotoxicity.

### CAY10594 shows therapeutic effects against APAP overdose-induced acute liver injury

**Figure 5:**
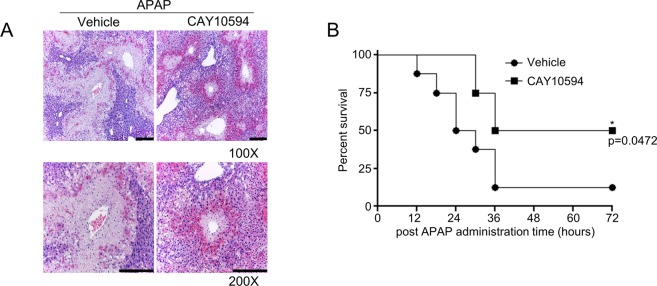
CAY10594 elicits therapeutic effects on APAP-induced acute liver injury. (**A**) After fasting for 16 h, APAP (500 mg/kg) was administered to mice. Either the vehicle (PBS containing 1% DMSO) or CAY10594 (8 mg/kg) was administered to the APAP-challenged mice at 3 h post APAP. The livers were isolated from the APAP-challenged mice at 12 h, and stained with hematoxylin and eosin (magnification, x100, x200) (**A**). The data are representative of two independent experiments with individual samples. Each experiment was performed in quintuplicates. Scale bar: 200 μm (**A**). (**B**) Survival rate was monitored from mice which were intraperitoneally injected with either the vehicle (PBS containing 1% DMSO) or CAY10594 (8 mg/kg) at 3 h after the APAP challenge (750 mg/kg). The survival rate was monitored every 6 h for 72 h. *P < 0.05 by log-rank test. Sample size: n = 8 (**B**).

Clinically, it is important to develop therapeutic agents against liver damage due to drug overdose. Therefore, we investigated whether CAY10594 shows therapeutic effects against APAP-induced acute liver injury by administering CAY10594 after the APAP challenge. Administration of CAY10594 (8 mg/kg) 3 h post APAP challenge markedly blocked APAP-induced liver damage, which was measured by histological analysis after hematoxylin and eosin staining (Fig. 5A). Mouse survival rate was also highly increased upon 8 mg/kg of CAY10594 administration at 3 h after APAP challenge, showing an 50% survival rate, respectively (Fig. 5B). Under the same experimental conditions, the vehicle administered group after APAP challenge showed a 12.5% survival rate at 72 h (Fig. 5B). These results strongly indicate that CAY10594 has therapeutic effects against APAP-induced acute liver injury.

## Discussion

PLD regulates lipid metabolism by hydrolyzing phosphatidylcholine to PA and choline^23^. PLD2 has been known to have a far higher basal activity than PLD1, and PLD2 mediates various unique protein interactions^24^. Moreover, PLD2 has different metabolic properties from PLD1, and most production of cyclic PA is dependent on PLD2 rather than PLD1^25^. Previously, it was reported that accumulation of PA can enhance the regeneration of liver after APAP-induced liver injury^17^. A separate report demonstrated that inhibition of PA production with FSG67, an inhibitor of glycerol 3-phosphate acyltransferase, did not affect the severity of APAP-induced acute liver injury at the injury phase^17^. However, in this study, we have observed preventive effects of CAY10594, showing 100% survival against the APAP-induced acute liver injury mice model (Fig. 1D). CAY10594 also induced strong therapeutic effects in APAP-challenged mice (Fig. 5). The results suggest an important role for PLD2, which crucially mediates APAP-induced liver injury. Because PLD2 has basal activity, administration of CAY10594 would block the generation of PA, the product of PLD2 enzymatic activity, in an experimental acute liver injury model. Therefore, it might reasonable to assume that the protective and therapeutic effects of CAY10594 in an acute liver injury model would be mediated by blocking the generation of PA. Based on our findings, we suggest that PA may have a pathological role in the disease progress of acute liver injury.

**Figure 6:**
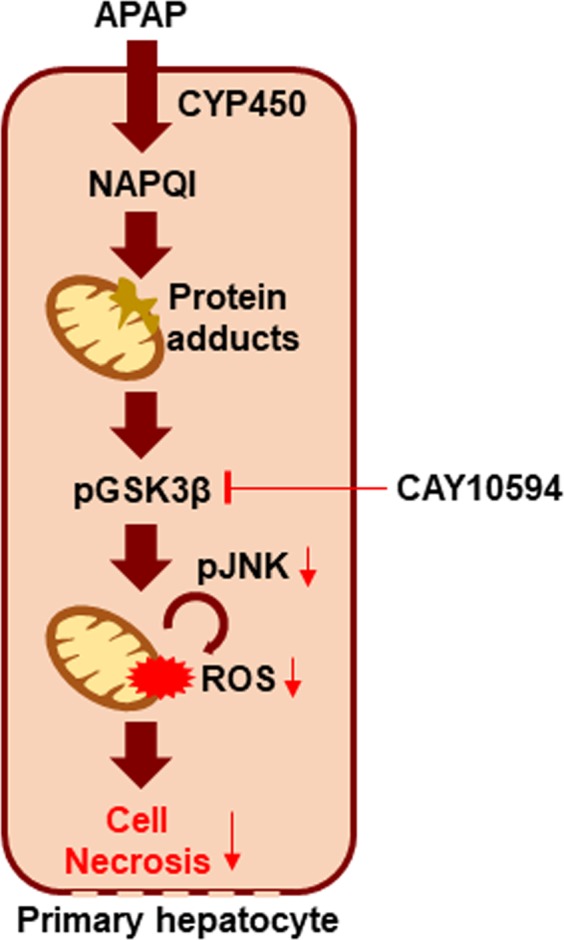
Working model of the therapeutic effects of CAY10594 against APAP-induced acute liver injury. CAY10594 blocks GSK-3β (Serine 9)/JNK phosphorylation in hepatocytes, eliciting therapeutic effects against APAP-induced acute liver injury model.

In APAP-induced acute liver injury, phosphorylation of JNK is a central player in inducing hepatic damage^26^. Sustained phosphorylation of JNK and translocation of JNK to the mitochondria exacerbates mitochondrial dysfunction, and causes hepatic necrosis. Moreover, previous reports already demonstrated that JNK inhibitors such as leflunomide, SP600125 or D-JNKI1 inhibited APAP-induced liver injury^27,28^. JNK phosphorylation can be differentially controlled in two different phases, the early and late phase. Phosphorylated-GSK-3β/JNK axis is a major source of APAP-induced liver injury at the very early phase. The early phase of JNK activation is regulated by GSK-3β, which can activate JNK through MEKK-1 or mixed lineage kinase-dependent pathways. On the other hand, the late phase of JNK activation is believed to mediate apoptosis signal-regulating kinase 1. Recently, p53 up-regulated modulator of apoptosis has been reported to be induced downstream of JNK and mediate APAP-induced necrosis and liver injury^29^. GSK-3β silencing has been reported to have beneficial effects against APAP-induced liver injury. Therefore, we tested whether PLD2 modulates phosphorylated-GSK-3β in the early phase following the APAP challenge. CAY10594 almost completely blocked GSK-3β phosphorylation when compared with vehicle administered mice (Fig. 2B). Our results suggest that CAY10594 may block JNK phosphorylation at the early phase by inhibiting GSK-3β phosphorylation (Fig. 6). At about 30 min to 6 h after the APAP challenge, NAPQI, the metabolite of APAP, induces the dysfunction of mitochondria and production of reactive oxygen species that initiate liver injury through JNK phosphorylation. Therefore, the regulation of reactive oxygen species is important in APAP-induced liver injury. These reactive oxygen species can be removed by GSH, but APAP overdose induces GSH depletion in an hour^30^. We found that CAY10594 prevents phosphorylation of JNK (Fig. 2B) and the regulation of GSK-3β phosphorylation, that might contribute to this mechanism (Fig. 2B). APAP challenge-induced GSH depletion was rapidly recovered upon CAY10594 administration (Fig. 2A). Since acute liver injury induced by APAP overdose has been reported to be mediated by the generation of excess oxidative stress^31^, it is important to regulate the expression of anti-oxidant genes in the liver to endure oxidative stress^32^. We also tested for a possible effect of CAY10594 on the regulation of anti-oxidant genes. The expression of several anti-oxidant genes, including *FGF21*, *Nrf2*, *HO-1* and *NQO1*, were highly expressed upon APAP overdose. However, anti-oxidant gene expression was not increased by CAY10594, with the expression levels remaining low compared to vehicle mice at 6 hours post APAP challenge (data not shown). Consequently, it appears that CAY10594-induced protective effects against APAP-induced liver injury is not mediated by upregulation of anti-oxidant genes. Collectively, our results suggest that CAY10594 may modulate early hepatopathology to prevent APAP-induced liver injury by rapid recovery of GSH levels without affecting anti-oxidant gene expression.

The pathological progress of acute liver injury caused by APAP administration can be mediated by early recruitment and activation of several immune cell types. This immune cell infiltration to the liver in a short period of time is regulated by several damaged-associated molecular patterns such as the S-100A families and HMGB-1 from hepatocytes^21^. Immune cells infiltrated into the liver can induce the secretion of inflammatory cytokines and chemokines, which contributes greatly to liver damage. In our study, we found that administration of CAY10594 did not affect the number of CD11b^hi^Ly6C^+^, CD11b^hi^Ly6G^+^, and CD11b^int^F4/80^+^ cells in APAP-challenged mice (Fig. 3C). These results suggest that PLD2 is not involved in immune cell population changes during APAP-induced liver damage.

If liver damage is suppressed at early time phases, inflammatory stimuli would not be secreted. TNF-α and IL-1β are known as major pro-inflammatory cytokines which play roles in DILI. TNF-α induces mitochondrial dysfunction via the TNF-receptor and induces hepatocyte death through caspase activation^33^. IL-1β is also an inflammatory cytokine that induces liver damage and is produced by activation of the inflammasome^34^. CAY10594 efficiently reduced secretion of pro-inflammatory cytokines and the CCL2 chemokine (Fig. 3A). Since we also observed that CAY10594 administration suppresses liver damage at the early phase, our results suggest that the protective effects of CAY10594 against acute liver injury would be directly mediated by regulation of hepatocytes but not by regulation of immune cell activity. Our findings of the direct inhibitory effects of CAY10594 on the hepatotoxicity and GSK-3β phosphorylation caused by APAP treatment (Fig. 4) further support our idea that CAY10594 may show protective effects against acute liver injury by modulating hepatocyte activity (Fig. 6). On the action mode of CAY10594, the inhibitor can inhibit GSK-3β/JNK axis to regulate ROS generation in mitochondria of hepatocytes (Fig. 6).

In APAP-induced liver injury, pharmacological treatment options are highly limited. For APAP overdose patients, a prescription of N-acetylcysteine is the only treatment since the 1970’s^35^. Metformin has also been revealed as a therapeutic candidate for APAP-induced liver injury, however, it must be administered at a concentration higher than 300 mg/kg like N-acetylcysteine. However, in our study, we demonstrate that CAY10594, which has been highlighted as being therapeutic without unacceptable clinical side effects^14^, has great therapeutic effects at a low dosage (8 mg/kg). Based on our current findings, we suggest that CAY10594 can be regarded as an important material for the development of therapeutic agents against APAP overdose-induced acute liver injury.

## Materials and Methods

### Animal study

C57BL/6 mice were purchased from DBL (Eumsung, Korea). All animal experiments were performed in accordance with the Korea Food and Drug Administration guidelines. Protocols were approved by the Animal Care and Use Committee, Sungkyunkwan University (Suwon, Korea). Mice were fasted for 16 hours before APAP injection. APAP (500 mg/kg) was administered with oral gavage in mice. CAY10594 (*N*-[2-(4-oxo-1-phenyl-1,3,8-triazaspiro[4,5]dec-8-yl)ethyl]-2-naphthalene carboxamide)^23^ was dissolved in 1% DMSO and intraperitoneally administered to mice 30 minutes prior to APAP injection for examining protective effects or after 3 hours from APAP challenge for investigating therapeutic effects of CAY10594.

### Histopathology and immunohistochemistry

Twelve hours after APAP (500 mg/kg) was administered with oral gavage in mice, the mice were euthanized, and their livers were isolated and fixed with 10% neutral buffered formalin. Liver tissues were embedded in paraffin and stained with hematoxylin and eosin for morphological analysis. The TUNEL assay was performed on paraffin-embedded tissue sections using a standard histological protocol. In brief, section slides were incubated using terminal deoxynucleotidyl transferase dUTP nick end labeling fluorescein for 1 hour at 37 °C in dark. The samples were washed 3 times in PBS for 3 minutes each and incubated with peroxidase conjugated anti-fluorescein antibody for 30 min at 37 °C in the dark. TUNEL positive cells were detected by diaminobenzidine solution. Liver histology and apoptotic cells were observed under a Leica ICC50 HD microscope.

### Measurement of serum AST and ALT

ALT and AST levels were measured using an ALT activity kit and AST activity assay kit (Sigma-Aldrich, St. Louis, MO, USA) according to the manufacturer’s instructions.

### GSH measurements

Liver tissue (33 mg) was homogenized with 5% metaphosphoric acid (500 μl) and centrifuged at 12,000 rpm for 10 minutes at 4 °C. GSH levels were measured from the supernatants using a glutathione (GSSG/GSH) detection kit according to the manufacturer’s instructions (Enzo Life Sciences Inc, Farmingdale, NY, USA). Total GSH levels were measured according to the net slope of the standard curve.

### Mitochondria isolation

Liver tissue (35 mg) was homogenized and used for the fractionation of mitochondria using a mitochondria isolation kit (Thermo Fisher Scientific^TM^, Waltham, MA USA) according to the manufacturer’s instructions.

### Western blot analysis

Homogenized liver samples or primary hepatocytes were lysed with RIPA buffer (150 mM NaCl, Tris-HCl pH 7.5, SDS 0.1%, Triton X-100 1%, EDTA 2 mM, 0.5% Na-deoxycholate) with proteinase inhibitor. Proteins were resolved by SDS-PAGE and transferred to a PVDF membrane, which was probed with primary antibodies against phospho-ERK, JNK, GSK-3β (Serine 9) and ERK, JNK, GSK-3β, COX IV (Cell Signaling Technology, Danvers, MA, USA) followed by incubation with secondary antibodies conjugated with horseradish peroxidase.

### Cytokine measurement

Blood samples were collected from acute liver injury mice 12 hours post-APAP treatment. Cells were removed from the harvested biofluids by centrifugation (12,000 rpm for 1 minute), and the levels of cytokines were measured by ELISA (eBioscience Inc., San Diego, CA, USA).

### Flow cytometry

Single cell suspensions of cells from collagenase treated liver samples were generated and blocked with FcγIII antibody and stained for CD11b (M1/70), Ly6G (1A8), Ly6C (HK1.4), F4/80 (BM8) on the cell surface. All those primary antibodies were purchased from eBioscience (San Diego, CA, USA). Cells were analyzed by flow cytometry and data were analyzed by FlowJo_V10.

### Isolation of primary hepatocytes and *in vitro* experiments with APAP treatment

Primary hepatocytes were isolated from wild-type mice following liver-specific perfusion with 50 ml of a buffer containing 66.7 mM NaCl, 6.7 mM KCl, 50 mM HEPES, 4.8 mM CaCl_2_ 2H_2_O, collagenase type IV. Cells were centrifuged at 500 rpm for 4 minutes. Primary hepatocytes were separated from dead cells and other cell types by Percoll gradient centrifugation (1,250 rpm for 5 minutes) and seeded into 6-well culture dishes (Thermo Fisher Scientific), and then cells were stimulated with 5~20 mM APAP with time dependency. Stimulated cells were fixed with 4% formaldehyde at room temperature for 10 minutes. Next, the cells were washed twice with 1 × PBS and stained with PI in 1 × PBS at room temperature for 10 minutes. PI positive cells were visualized with a fluorescence microscope (KI-2000F, Korea Lab Tech, Seongnam, Korea) and images were analyzed with OptiView 3.7.

### Cytotoxicity assay

Cytotoxicity was assessed by detection of the enzyme lactate dehydrogenase (LDH) in the supernatant of the cell culture. The assay was performed using the Cytotoxicity Detection Kit (Promega, Madison, WI, USA) according to the manufacturer’s protocol. The LDH concentration in 50 µl of the cell culture supernatant was determined at a wavelength of 490 nm. Cells treated with lysis solution served as a reference for the maximum possible LDH release (100%, high control). The relative LDH release of a given sample is then defined as the ratio of LDH measured in the supernatant of the sample and the high control value with LDH values under 10% regarded as a nontoxic effect level.

### Statistical analysis

Results were evaluated using GraphPad prism software. Statistical analysis was performed by Student’s *t*-test. All results are expressed as the mean ± SEM. Survival data were analyzed using the log-rank test. A *P* value ≤ 0.05 was considered statistically significant.

## References

[1] Larson AM (2005). Acetaminophen-induced acute liver failure: results of a United States multicenter, prospective study. Hepatology.

[2] Wilcox CM, Cryer B, Triadafilopoulos G (2005). Patterns of use and public perception of over-the-counter pain relievers: focus on nonsteroidal antiinflammatory drugs. J. Rheumatol..

[3] Placke ME, Ginsberg GL, Wyand DS, Cohen SD (1987). Ultrastructural changes during acute acetaminophen-induced hepatotoxicity in the mouse: a time and dose study. Toxicol. Pathol..

[4] Kon K, Kim J-S, Jaeschke H, Lemasters JJ (2004). Mitochondrial permeability transition in acetaminophen-induced necrosis and apoptosis of cultured mouse hepatocytes. Hepatology.

[5] Mitchell JR (1973). Acetaminophen-induced hepatic necrosis. I. Role of drug metabolism. J. Pharmacol. Exp. Ther..

[6] Xu JJ, Hendriks BS, Zhao J, de Graaf D (2008). Multiple effects of acetaminophen and p38 inhibitors: towards pathway toxicology. FEBS Lett..

[7] Han Derick, Shinohara Mie, Ybanez Maria D., Saberi Behnam, Kaplowitz Neil (2009). Signal Transduction Pathways Involved in Drug-Induced Liver Injury. Handbook of Experimental Pharmacology.

[8] Hanawa N (2008). Role of JNK translocation to mitochondria leading to inhibition of mitochondria bioenergetics in acetaminophen-induced liver injury. J. Biol. Chem..

[9] Shinohara M (2010). Silencing glycogen synthase kinase-3beta inhibits acetaminophen hepatotoxicity and attenuates JNK activation and loss of glutamate cysteine ligase and myeloid cell leukemia sequence 1. J. Biol. Chem..

[10] Oude Weernink PA, López de Jesús M, Schmidt M (2007). Phospholipase D signaling: orchestration by PIP2 and small GTPases. Naunyn. Schmiedebergs. Arch. Pharmacol..

[11] Jenkins GM, Frohman MA (2005). Phospholipase D: a lipid centric review. Cell. Mol. Life Sci..

[12] Peng X, Frohman MA (2012). Mammalian phospholipase D physiological and pathological roles. Acta Physiol. (Oxf)..

[13] Park JB (2012). Phospholipase signalling networks in cancer. Nat. Rev. Cancer.

[14] Frohman MA (2015). The phospholipase D superfamily as therapeutic targets. Trends Pharmacol. Sci..

[15] Ghim J (2014). Endothelial deletion of phospholipase D2 reduces hypoxic response and pathological angiogenesis. Arterioscler. Thromb. Vasc. Biol..

[16] Lee SK (2015). Phospholipase D2 drives mortality in sepsis by inhibiting neutrophil extracellular trap formation and down-regulating CXCR2. J. Exp. Med..

[17] Lutkewitte AJ (2018). Lipin deactivation after acetaminophen overdose causes phosphatidic acid accumulation in liver and plasma in mice and humans and enhances liver regeneration. Food Chem. Toxicol..

[18] Jaeschke H, McGill MR, Williams CD, Ramachandran A (2011). Current issues with acetaminophen hepatotoxicity–a clinically relevant model to test the efficacy of natural products. Life Sci..

[19] Liu F-C, Lee H-C, Liao C-C, Li AH, Yu H-P (2016). Tropisetron Protects Against Acetaminophen-Induced Liver Injury via Suppressing Hepatic Oxidative Stress and Modulating the Activation of JNK/ERK MAPK. Pathways. Biomed Res. Int..

[20] Ong MMK, Latchoumycandane C, Boelsterli UA (2007). Troglitazone-induced hepatic necrosis in an animal model of silent genetic mitochondrial abnormalities. Toxicol. Sci..

[21] Martin-Murphy BV, Holt MP, Ju C (2010). The role of damage associated molecular pattern molecules in acetaminophen-induced liver injury in mice. Toxicol. Lett..

[22] Blazka ME, Wilmer JL, Holladay SD, Wilson RE, Luster MI (1995). Role of proinflammatory cytokines in acetaminophen hepatotoxicity. Toxicol. Appl. Pharmacol..

[23] Scott SA (2009). Design of isoform-selective phospholipase D inhibitors that modulate cancer cell invasiveness. Nat. Chem. Biol..

[24] Hancock, J. F. NEWS and VIEWS PA promoted to manager. **9**, 615–617 (2007). 10.1038/ncb0607-615 17541412

[25] Tsukahara T (2010). Article Phospholipase D2-Dependent Inhibition of the Nuclear Hormone Receptor PPAR g by Cyclic Phosphatidic Acid. Mol. Cell.

[26] Jaeschke H (2016). Mechanisms of Acetaminophen Hepatotoxicity: Do We Need JNK for Cell Death?. Gastroenterology.

[27] Latchoumycandane C, Goh CW, Ong MM, Boelsterli UA (2007). Mitochondrial protection by the JNK inhibitor leflunomide rescues mice from acetaminophen-induced liver injury. Hepatology.

[28] Henderson NC (2007). Critical role of c-jun (NH2) terminal kinase in paracetamol- induced acute liver failure. Gut..

[29] Chen Dongshi, Ni Hong‐Min, Wang Lei, Ma Xiaowen, Yu Jian, Ding Wen‐Xing, Zhang Lin (2019). p53 Up‐regulated Modulator of Apoptosis Induction Mediates Acetaminophen‐Induced Necrosis and Liver Injury in Mice. Hepatology.

[30] Woolbright BL, Jaeschke H (2017). Role of the Inflammasome in Acetaminophen-induced Liver Injury and Acute Liver Failure. J. Hepatol..

[31] Jaeschke H (1990). Glutathione disulfide formation and oxidant stress during acetaminophen-induced hepatotoxicity in mice *in vivo*: the protective effect of allopurinol. J. Pharmacol. Exp. Ther..

[32] Ye D (2014). Fibroblast growth factor 21 protects against acetaminophen-induced hepatotoxicity by potentiating peroxisome proliferator-activated receptor coactivator protein-1α-mediated antioxidant capacity in mice. Hepatology.

[33] Han D (2013). Regulation of drug-induced liver injury by signal transduction pathways: Critical role of mitochondria. Trends Pharmacol. Sci..

[34] Williams CD (2011). Role of the Nalp3 inflammasome in acetaminophen-induced sterile inflammation and liver injury. Toxicol. Appl. Pharmacol..

[35] Du K, Ramachandran A, Jaeschke H (2016). Oxidative stress during acetaminophen hepatotoxicity: Sources, pathophysiological role and therapeutic potential. Redox Biol..

